# Texture recognition based on multi-sensory integration of proprioceptive and tactile signals

**DOI:** 10.1038/s41598-022-24640-5

**Published:** 2022-12-15

**Authors:** Behnam Rostamian, MohammadReza Koolani, Pouya Abdollahzade, Milad Lankarany, Egidio Falotico, Mahmood Amiri, Nitish V. Thakor

**Affiliations:** 1 Medical Biology Research Center, Institute of Health Technology, Kermanshah University of Medical Sciences, Kermanshah, Iran; 2 Krembil Research Institute, University Health Network (UHN), Toronto, ON Canada; 3 Institute of Biomedical Engineering and Department of Physiology, University of Toronto, Toronto, Canada; 4 The BioRobotics Institute, Scuola Superiore Sant’Anna, Pontedera, Italy; 5 Medical Technology Research Center, Institute of Health Technology, Kermanshah University of Medical Sciences, Parastar Ave., Kermanshah, Iran; 6 Department of Biomedical Engineering, Johns Hopkins University, Baltimore, MD USA; 7 Department of Biomedical Engineering, National University of Singapore, Singapore, Singapore

**Keywords:** Sensory processing, Biomedical engineering

## Abstract

The sense of touch plays a fundamental role in enabling us to interact with our surrounding environment. Indeed, the presence of tactile feedback in prostheses greatly assists amputees in doing daily tasks. In this line, the present study proposes an integration of artificial tactile and proprioception receptors for texture discrimination under varying scanning speeds. Here, we fabricated a soft biomimetic fingertip including an 8 × 8 array tactile sensor and a piezoelectric sensor to mimic Merkel, Meissner, and Pacinian mechanoreceptors in glabrous skin, respectively. A hydro-elastomer sensor was fabricated as an artificial proprioception sensor (muscle spindles) to assess the instantaneous speed of the biomimetic fingertip. In this study, we investigated the concept of the complex receptive field of RA-I and SA-I afferents for naturalistic textures. Next, to evaluate the synergy between the mechanoreceptors and muscle spindle afferents, ten naturalistic textures were manipulated by a soft biomimetic fingertip at six different speeds. The sensors’ outputs were converted into neuromorphic spike trains to mimic the firing pattern of biological mechanoreceptors. These spike responses are then analyzed using machine learning classifiers and neural coding paradigms to explore the multi-sensory integration in real experiments. This synergy between muscle spindle and mechanoreceptors in the proposed neuromorphic system represents a generalized texture discrimination scheme and interestingly irrespective of the scanning speed.

## Introduction

The human sense of touch plays a key role in the somatosensory system, enabling us to interact with the surrounding world. In conjunction with tactile sensation, proprioception allows us to perform daily manipulation tasks that provide perception and interaction with objects. To respond to the external stimuli delivered to glabrous skin, the primary mechanoreceptors, namely, Merkel cells, Meissner, and Pacinian corpuscles transmit information to the other levels in tactile processing pathways^1^. To discriminate the texture of objects, we palpate fingers across the surface with variable scanning speed and contact force, leading to corresponding spatial and temporal skin responses. Moreover, tactile receptors’ temporal spiking patterns encode fine textural features depending on the scanning speed^2^. Therefore, variations in scanning speed are expected to lead to associated changes in the firing rate of afferents with a proportional relationship. Despite many changes in sensory receptors' activity patterns, the nervous system attains stable perceptual representations of textures^2^ and indeed our perception of the attributes remains stable at variable scanning speeds^3^. This texture perception, irrespective of palpation speed and mechanoreceptors’ dependency, implies that there should be some other mechanism contributing to this ability that exploits information about scanning speeds during active touches^4^.

Active touch involves activating the cutaneous, kinesthetic, and proprioceptive senses, which assist us in perceiving scanning parameters and discriminating object qualities, whereas passive touch activates only the operation of the cutaneous receptors of the glabrous skin^5^. In other words, active touch evokes more distributed brain activity in areas outside the somatosensory domain than passive touch, perhaps due to motor control ability. The motor control ability is responsible for regulating or directing the essential mechanisms of movement, including the neurological, musculoskeletal, and sensory/perceptual systems^5,6^.

Inspired by the biological mechanisms in neuroscience, neuromorphic engineering is an interdisciplinary field that aims to emulate neuronal activities with the aid of electronic devices to encode external stimuli as a sequence of action potentials (spike trains)^7,8^. In particular, a neuromorphic system translates sensory information into a pattern of spikes for the communication of sensors with the brain model simulations^9^. The representation of the sensor’s data by spikes possesses an anti-noise capability and offers a reliable propagation^10^. Recently, different approaches have been taken by using advanced electronic skin (e-skin) and artificial fingertips through the spiking activity of artificial mechanoreceptors^11^. These bio-inspired systems attempt to provide the skin-like sensory capability to prosthetic/robotic hands to deliver valuable information about joints (proprioception) and estimate realistic force to grasp objects^12^. In other words, advances in neuromorphic research allow the development of artificial mechanoreceptors to produce spike trains^9^. Prior research studies have discriminated roughness using artificial tactile sensors or artificial fingertips^10,13^. Oddo et al.^13^ have reported that the frequency of the sensor vibrations is shifted systematically to higher frequencies with increasing scanning speed, and vice versa. This increment and decrement in scanning speed lead to the wrong recognition of the palpated textures. Rongala. et al.^14^ have implemented a two-layer spiking neuronal network that learns tactile features with varying conditions for discriminating textures without providing velocity feedback or sensor fusion capability. The same texture-palpation test was repeated with several velocities to discriminate various textures under different scanning speeds. Liu et al.^15^ have modeled cutaneous mechanoreceptors of human glabrous skin for discriminating stimulus, based on the response time interval between adjacent sensor units and the principal frequency of vibration. Oddo et al.^16^ have used a MEMS sensor to discriminate roughness in active touch, which was done by using the phase difference between two adjacent taxels. Bouganis and Shanahan^17^ have used encoders to provide feedback to iCub (a research-grade humanoid robot^18^) arm joints using the neuron’s firing rate for encoding the current angle of joints.

In humans, there is multi-dimensional sensory feedback, where the fusion of the tactile and proprioception sensory data assists us in sensing the scanning speed during tactile manipulation tasks^14^. However, such observation has not been artificially investigated nor recruited to explore the results of synergy between muscle spindles and tactile receptors in texture discrimination with variable scanning speeds. Indeed, many studies have disregarded scanning speed variations in this field by applying predetermined constant speeds^10,19^ which limits the generalizability of these findings under dynamic conditions. In this research, a hydro-elastomer sensor was fabricated to mimic the muscle spindle organ. In this case, we investigated the role of muscle spindles in texture discrimination during active touch. It should be pointed out that the proprioceptive system mostly depends on the length and velocity of the muscle, in which the muscle spindle plays an important role^20^. Moreover, three tactile mechanoreceptors were employed for distinguishing multiple fine naturalistic textures under varying speed condition in conjunction with artificial proprioceptive feedback. To the best of our knowledge, this is the first example of the integration of proprioceptive sensory feedback with three types of artificial mechanoreceptors using a soft biomimetic fingertip for recognition of naturalistic texture independent of the scanning speed.

## Background

**Figure 1:**
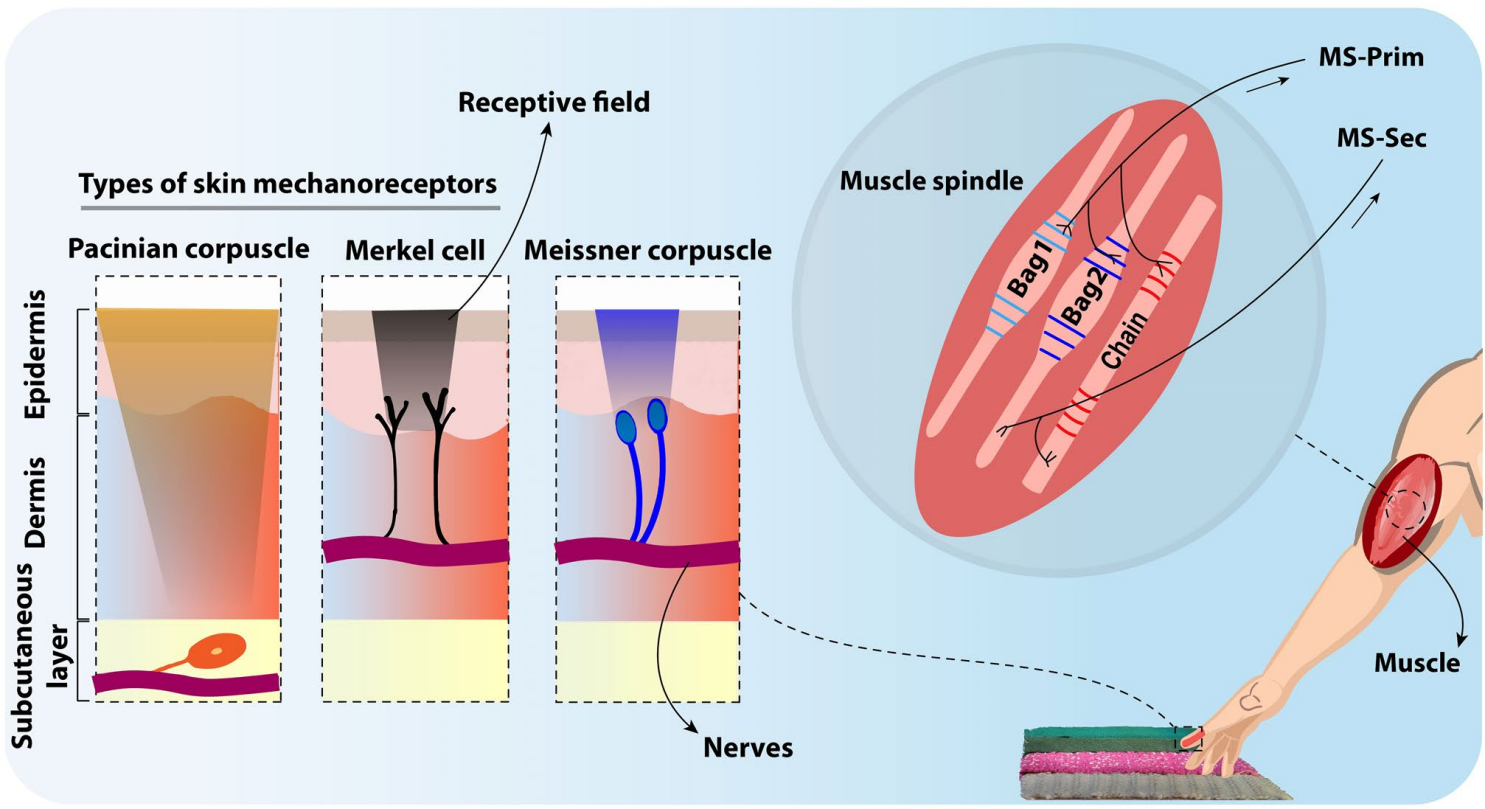
The schematic of the biological sensory system. Left, illustration of tactile mechanoreceptors with their receptive fields within human skin to sense surface details. Right, the muscle spindle organ with three types of intrafusal fibers that respond through primary (MS-Prim) and secondary (MS-Sec) afferents for encoding the position and velocity of the muscle.

Human beings perceive textures with various mechanoreceptors to sense spatial details of texture under variable exploring parameters such as scanning speed during daily tasks^2,21^. To address this question of how the human's sensory perception encodes the texture characteristics, recent studies showed that the tactile texture recognition for fine surfaces relies mainly on vibration-sensitive afferents^22^ such as fast adapting mechanoreceptors (Meissner (RA-I) and Pacinian (RA-II) corpuscles) and slowly adapting afferents (Merkel discs (SA-I)) for coarse textures. Figure 1 illustrates three types of skin mechanoreceptors, Merkel and Meissner mechanoreceptors are in the dermis layer of skin and close to the epidermis layer. Merkel cells (SA-I) respond to static cues like skin indentation (contact force) and spatial characteristics of coarse textures^20^. Furthermore, Meissner corpuscles (RA-I) sense low-frequency stimuli and elicitation of skin. Both SA-I and RA-I receptive fields are too big to respond to small elements of textures (fine textures)^2^. However, Pacinian corpuscles (RA-II) in the deeper layer of skin (subcutaneous layer) are sensitive to high-frequency stimuli and encode small elements by temporal characteristics of the spiking patterns^23^. Meissner (RA-I), and Pacinian (RA-II) corpuscles convey fine texture characteristics although their spiking patterns are sensitive to texture features and scanning speed^20^. Nevertheless, the question is how neural mechanisms tolerate speed dependency to achieve stable texture discrimination. Perhaps, human sensory fusion can construct multifaceted sensory perception during active touch; information from muscle spindles^9^ and Ruffini endings contribute to estimating scanning speed as the proprioceptive feedback^14,24^. Skeletal muscles play a vital role in maintaining posture and balance^25^. They assist us in moving and performing daily tasks. Muscle afferents respond to unexpected loads or obstacles to trigger the appropriate adjustments^26^. Indeed, proprioception and kinesthesia sensing are crucial when performing voluntary movements to provide unconscious peripheral feedback (scanning speed and limb position) during posture and locomotion^26^. The primarily responsible receptors for proprioception sensing are muscle spindles, Golgi tendon organs, and free nerve endings in the joint capsule^27^. The proprioceptive system depends on the length and velocity of the muscle, and hence muscle spindle is the main foundation of proprioceptive feedback^9^. This specific type of fiber lies along extrafusal fibers and provides proprioceptive information about the muscle states within the belly of a skeletal muscle^9^. Their failure produces some impairments in humans^9,28–30^. Furthermore, some studies have reported that the muscle spindles directly play a key role in roughness discrimination as vibration-sensitive sensors during low contractions^21,31^. There are two static and dynamic subtypes of muscle spindles, where dynamic and static fibers respond to changes in length and absolute length of the spindle, respectively. The muscle spindle contains three types of intrafusal fibers: bag1, bag2, and chain that receive several fusimotor inputs (gamma static and dynamic) and muscle length^32^, as shown in Fig. 1. The muscle spindle organ generates two outputs: primary (MS-Prim) and secondary (MS-Sec) afferent activity, in which, MS-Sec is sensitive to muscle's length and MS-Prim is sensitive to both length and velocity of muscle^9,32^. Several models of muscle spindles afferents have been represented. More complex spindle models have been developed to simulate the afferent fibers' firing rate as a polynomial function of the muscle stretching and its speed^27,29^. In the present work, we employ a bio-inspired mechanism for translating proprioceptive feedback that implements a computational model of muscle spindle activity. In particular, the employed muscle spindle model is based on model proposed by Mileusnic et al.^32^, including fusimotor activation, primary and secondary afferent activities^32,34^. The Mileusnic’s model was comprehensively explained in^9,32^.

### Approach

**Figure 2:**
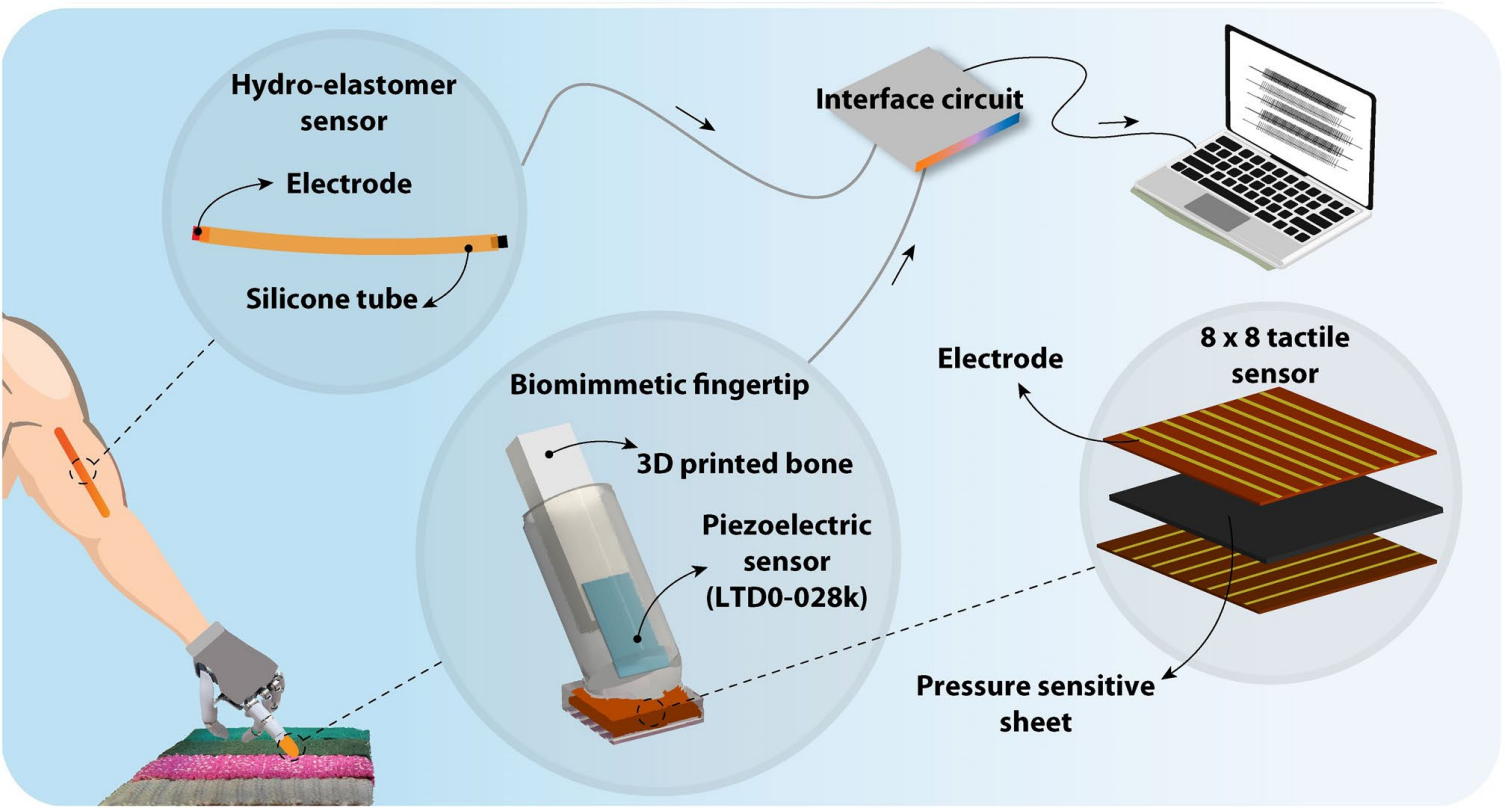
The schematic of the proposed multi-channel neuromorphic system. The artificial sensors on the prosthetic finger transduces contact and motion events during texture scanning to spiking responses. The detailed structure of the biomimetic fingertip and hydro-elastomer sensor are also shown. The 8 × 8 tactile sensor for mimicking SA-I/RA-I afferents to sense static/dynamic skin indentation. The piezoelectric sensor was embedded in the inner layer of the soft fingertip, mimicking Pacinian corpuscles. The hydro-elastomer sensor was used to emulate the muscle spindles.

We present a sensor-integration approach based on the human sensory systems, enabling texture recognition by a robotic finger. The proposed biomimetic fingertip consisted of an 8 × 8 array tactile sensor and a piezoelectric sensor for mimicking three biological tactile mechanoreceptors. The 8 × 8 array tactile sensor is a pressure-sensitive sensor for mimicking SA-I/RA-I afferents to sense static/dynamic skin changes while scanning textures. Moreover, the piezoelectric sensor was embedded within the inner layer of the soft biomimetic fingertip, mimicking Pacinian corpuscles in the deeper layer of skin with broader receptive fields to detect elicited high-frequency vibrations of fine textures. Then a highly stretchable proprioception sensor was utilized to emulate the muscle spindle with its afferents, namely, Primary (MS-Prim) and Secondary (MS-Sec). The proposed multi-channel neuromorphic system that resembles human skin mechanoreceptors and muscle spindles is illustrated in Fig. 2. The proposed sensory system enabled multisensory integration with a simple structure and uniform sensing elements to discriminate objects’ texture, sense manipulation speed, and position. The biomimetic fingertip comprises two sensing components: a commercial LTD0-028 K piezoelectric sensor and an 8 × 8 array tactile sensor (Fig. 2). Moreover, the 8 × 8 array tactile sensor consists of a pressure-sensitive sheet sandwiched between two layers of the flexible printed circuit board (PCB), See more details in Material and Methods section (Fig. 2). It is located in the outer layer of the biomimetic fingertip to mimic both Merkel cell and Meissner corpuscle. The hydro-elastomer sensor is used as a proprioception sensor to mimic the biological muscle spindle organ **(**Fig. 2**)**. The fabricated proprioception sensor is highly stretchable (100% strain), transparent, lightweight, inexpensive, and easy to fabricate with off-the-shelf materials. In this way, the raw data from different sensors are collected, preprocessed, and fed to the corresponding neurons and afferents (Materials and Methods). Then, using the rate coding algorithm, the spike information was extracted and used as the inputs of the machine learning classifiers to discriminate different textures. Following this section, two independent experiments have been accomplished for evaluating the employed sensory system in variable conditions.

## Results

### Innervation technique for SA-I and RA-I afferents

**Figure 3:**
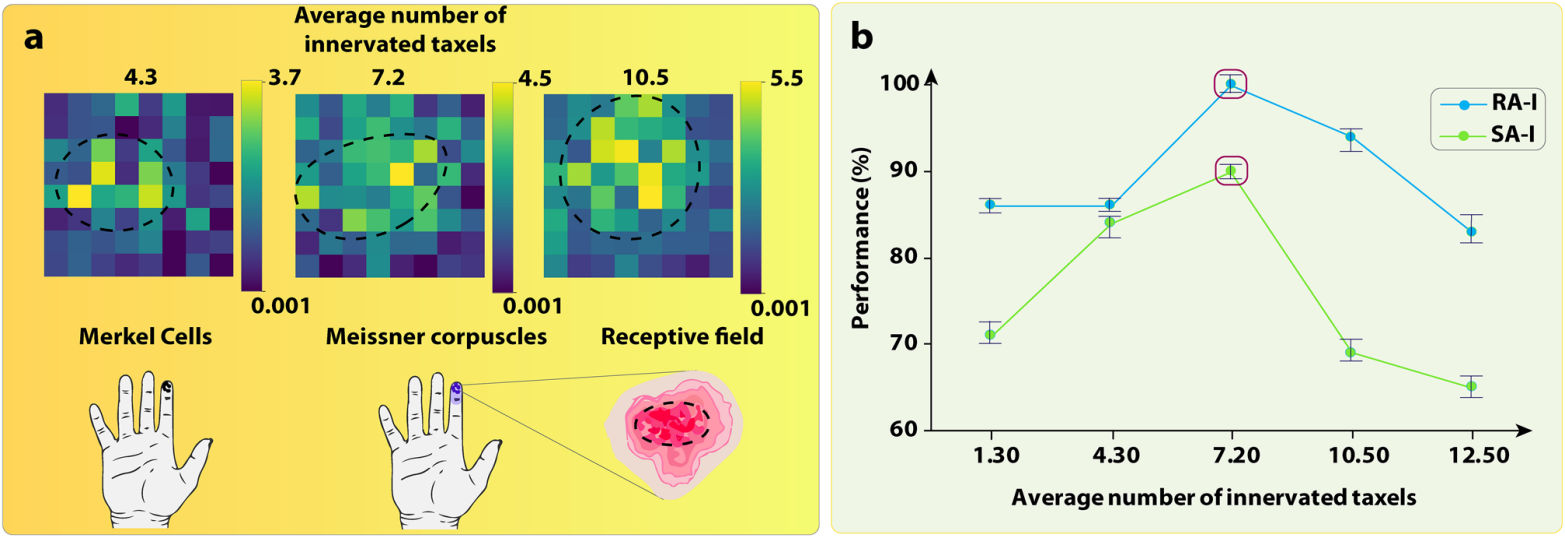
The effect of innervation on the robotic data classification. (**a**)-up, The three innervation samples of the receptive field for both SA-I and RA-I afferents with the different average number of innervated taxels by one afferent. The black dashed circles illustrate highly sensitive zones. The heatmap illustration was used to demonstrate the weight of each taxel. (**a**)-down, The receptive field of two classes of mechanoreceptors, Merkel and Meissner in which Meissner's receptive fields are shown. (**b**) Classification performance of SA-I and RA-I afferents for texture recognition (S1 to S6) when the receptive field size is changed. The Firing rate algorithm was used right after the sensor started to scan the surface.

Fine textures are classified in terms of vibrations, whereas coarse textures are classified in the spatial pattern during indentation^35^. The SA-I/RA-I afferents innervate the 64 taxels of the 8 × 8 tactile sensor similar to the innervation technique used in^36^. Indeed, spatially nearby taxels of the 8 × 8 tactile sensor are connected to one afferent with different weights, creating complex receptive fields (RF) that can encode contact force and stimuli details which means that the afferent obtains tactile signals from multiple taxels (Fig. 3a). Based on the experimental observation, the first-order neurons in the tactile sensory pathway branch in the skin and form many transduction sites^37^. Figure 3a illustrates 64 taxels (sensing elements) with different weights in which brighter taxels are innervated by more afferents and darker ones are innervated by one afferent with low weights (the lowest value is 0.001). In the other words, increasing the average number of innervated taxels by one afferent leads to the creation of taxels with higher weights (bright colors) and vice versa. Additionally, the innervation technique reduces the cost and processing time of 64 channels and needs fewer afferents to be implemented on hardware, leading to lower power consumption^36^. When a few taxels are activated, due to the overlaps, more afferents with different activity patterns provide information about manipulation. This random innervation produces diverse spiking responses due to overlaps in receptive fields. Various innervation patterns were investigated to shed light on the receptive field's contributions to encode an object's spatial frequency. Some examples of receptive fields with different innervation arrays have been demonstrated in Fig. 3a, in which brighter colors show a higher weight and hence resulting in a higher instantaneous firing rate.

The biomimetic fingertip scanned 6 textures (S1-S6, see Material and Methods section) by the fastest scanning speed (90 mm/s) and weakest touch (indentation) while only the 8 × 8 tactile sensor data has been recorded and converted to spike trains for focusing on the average number of innervated taxels (the biomimetic fingertip signals were collected offline). In this section, we focus on the innervation technique under the worse conditions to find the best average number of innervated taxels for texture discrimination. The details about objects, experimental setup, and neuromorphic models are explained in the Material and Methods. For examining the biomimetic SA-I/RA-I afferents responses, the firing rate algorithm^38^ of 36 digital afferents (24 RA-I and 12 SA-I) with different sizes of innervation are extracted as the input of the K-nearest neighbor (KNN) classifier. Figure 3b shows the classification accuracy of discriminating 6 fine naturalistic textures (S1-S6) when a different average number of taxels is innervated by one afferent, including 1.3, 4.3, 7.2, 10.5, and 12.5 in separate experiments. Decreasing the average number of innervated taxels leads to the creation of many taxels with low gains which are not highly sensitive to convey spatial information of the scanned texture just as higher gain taxels. Conversely, increasing the high gain taxels creates SA-I/RA-I afferents with similar innervated taxels, leading to lower performance due to similar information. For each classification, all data were randomly split into the training set and the test set with an 8:2 ratio. Then, all data were divided into 5 subsets to employ fivefold cross-validation. Every subset was used as the validation set and the remaining subsets formed the training set. Finally, the average performance across all 5 trials for each subset was computed. As it is obvious from the results shown in Fig. 3b, a receptive field with the 7.2 average number of innervated taxels is the efficient innervation method that gives the highest classification performance with the accuracy of 99.9% (k = 1) for RA-I and 88% (k = 3) for SA-I.

### Texture discrimination under variable scanning speeds

**Figure 4:**
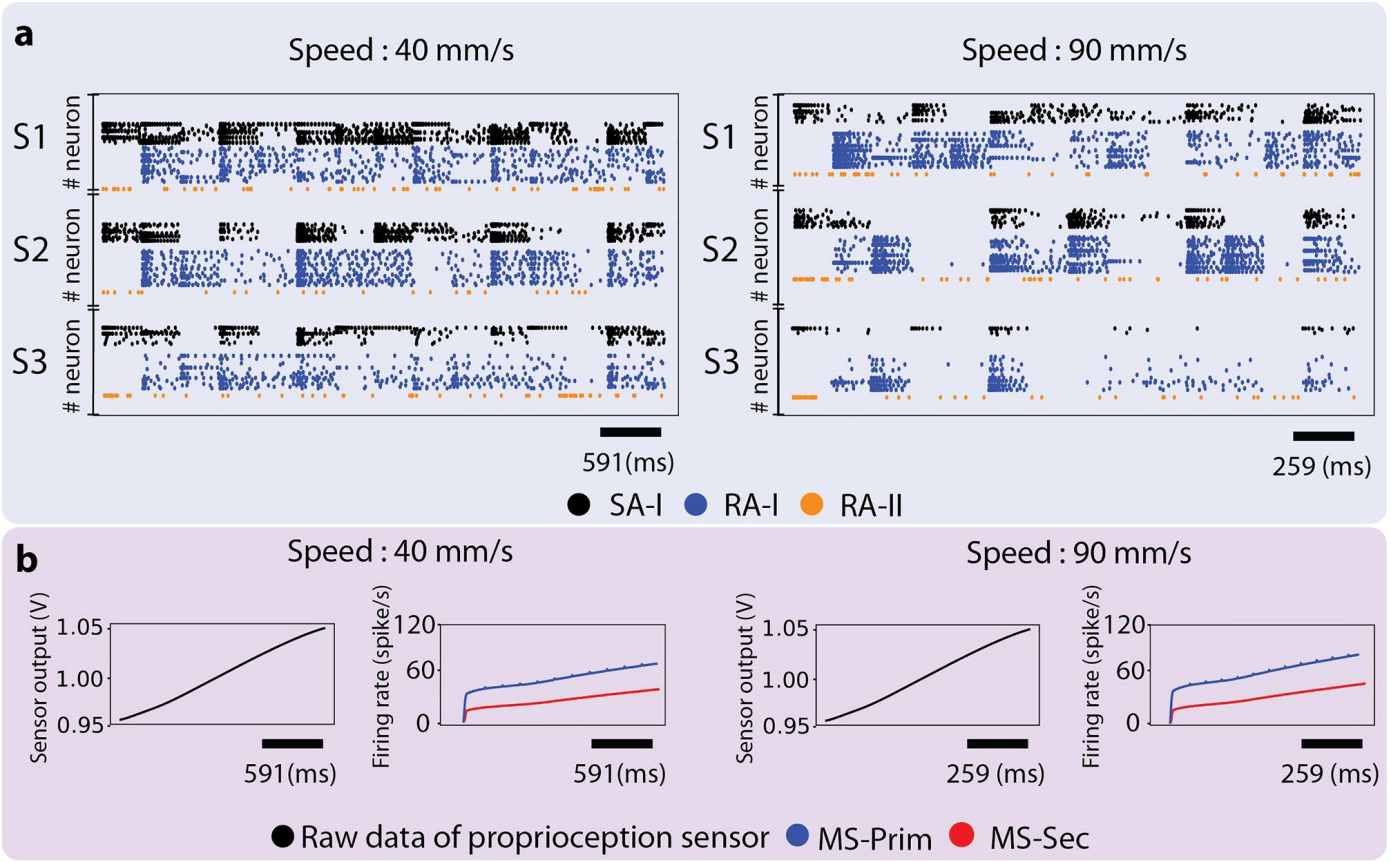
The responses of 12 SA-I, 24 RA-I, and 1 RA-II afferent in conjunction with muscle spindles afferents (MS-Prim and MS-Sec) for two scanning speeds (40 and 90 mm/s) and 3 surfaces (S1, S2, and S3). (**a**) The raster plot of the skin’s mechanoreceptors (spike train) for SA-I (Black), RA-I (Blue), and RA-II (Orange) for different surfaces. (**b**) The mapped length of the proprioception sensor and spike rate of muscle spindle afferents (MS-Prim (Blue) and MS-Sec (Red)) are shown (firing rates during the manipulation process), respectively.

This research offers an innovative neuromorphic approach to mimicking human sensory fusion by emulating muscle spindles and skin mechanoreceptors except for Ruffini endings (SA-II) because they are not responsible for the texture perception, hence, irrelevant to the scope of the current study. This technical approach artificially replicates the firing responses of the SA/RA afferents and the muscle spindle's primary (MS-Prim)/secondary (MS-Sec) afferents. Muscle spindles dominate proprioceptive and kinesthetic sensing of rapid and accurate hand movements more than other muscle receptors in human muscles^33^. In this work, a proprioception sensor is used to mimic muscle spindle organs for converting the position and velocity of the fingertip into voltages. Then, the sensor output voltages are used as the inputs of the mathematical model of the muscle spindle for evaluating primary and secondary afferents’ responses during active touch. Moreover, the soft biomimetic fingertip sensors’ data are collected, preprocessed, and fed to the corresponding artificial neurons and afferents. Finally, the spiking responses of all artificial afferents are used as the inputs of the machine learning classifiers to discriminate different textures across scanning speeds. All following results were taken from the experiments that the biomimetic fingertip scanned 10 naturalistic textures (S1 to S10), with 6 different scanning speeds ten times (40, 50, 60, 70, 80, 90 mm/s) which is well within the range commonly used in related neurophysiologic studies from 10 mm/s to 150 mm/s and human natural exploratory movements^39^. All the experimental data were collected without using any controller for monitoring normal contact force. Just indentation has been controlled for every 600 experiments (6*10*10, speed*texture*repetition). Moreover, the proprioception sensor was used to provide proprioceptive feedback during manipulations. The palpation speeds were categorized into three classes (low, medium, and high) to provide human-like speed detection and decrease class labels of employed classifiers. The system could accurately recognize many complex and similar textures with various dynamic conditions (scanning speeds). To mimic the biological mechanoreceptors' firing patterns, the outputs of an 8 × 8 tactile sensor and a piezoelectric sensor were converted into spike trains. The voltage of each sensor is used as the input current to the Izhikevich model^40^ (more details in the Neuromorphic Encoding section). From a neuromorphic point of view, changes in the sensing conditions induce differences in the firing patterns, but spike trains mainly reflect the spatial structure of the texture^14^. Similarly, we observe that changing the sliding speed for the same texture resulted in a coherent spike train structure occurring over different time scales (hence the higher firing rate for faster sliding). In Fig. 4, the responses of muscle spindle receptors and tactile afferents are shown. As mentioned in the Material and Methods, the outputs of the muscle spindle mathematical model are already firing rates of the two muscle spindle’s afferents, and hence their responses are not converted to the spike trains again^9,32^. However, the tactile afferent responses are illustrated as the spike trains during the manipulation process. An increase in the scanning speed causes the tactile sensors to detect higher-frequency vibrations induced by the surfaces^13^, resulting in the higher firing rates of the three mentioned tactile mechanoreceptors^2^ (Fig. 4a). These results show that the spike rate of the emulated spindles is increased with the stretching of the proprioception sensor (See Material and Methods). Next, we evaluate how sensing conditions affect the neuromorphic sensor's response and encoding of tactile information. The spike rate of tactile mechanoreceptors is increased with the increase in the sliding speed, across three textures (S1, S2, and S3 in Material and Method sections) (Fig. 4). Crucially, changes in the sensing conditions induced variations in the firing patterns of tactile mechanoreceptors resulting in decreasing the accuracy of the texture discrimination. As apparent from the responses of RA mechanoreceptors in Fig. 4a**,** the spike trains are quite sensitive to the scanning speed, which confuses the classifier; however, muscle spindle afferents’ responses (Fig. 4b) facilitate the clearance of this confusion and better distinguishing of these textures. In other words, the texture discrimination in conjunction with the proprioception sensor yields better accuracies.

**Figure 5:**
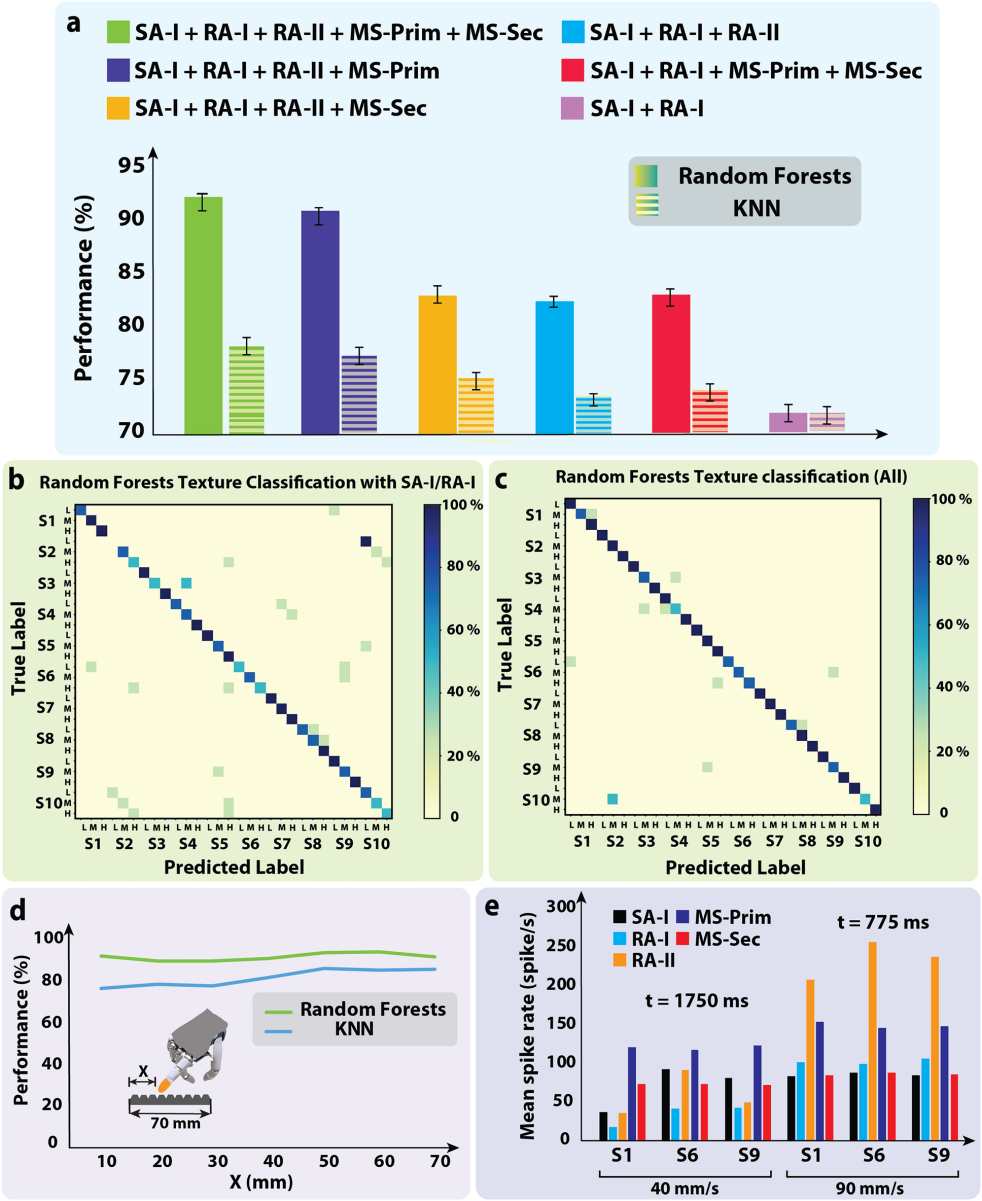
Contribution of the skin mechanoreceptors and muscle spindles in texture discrimination. (**a**) Classification accuracy for each SA-I/RA-I/RA-II/MS-Prim/MS-Sec population and their combination for all textures based on the spike rate algorithm for KNN and RF classifiers. (**b**) Confusion matrix for classifying 10 textures with 6 scanning speeds only for SA-I and RA-I afferents using Random forests classifier. (**c**) Confusion matrix for classifying 10 textures with 6 scanning speeds for integration of all mechanoreceptors and muscle spindles using Random forests classifier. (**d**) Classification performance for different lengths of finger’s displacement on the surface for KNN and Random forests classifiers. (**e**) The mean spike rate of mechanoreceptors and muscle spindle afferents during the response to three naturalistic surfaces (S1, S6, and S9) under two scanning speeds (slowest, 40 mm/s, and fastest, 90 mm/s).

To analyze the spiking activity of the afferents, we used the firing rate algorithm to extract reliable features as the inputs of the machine learning classifiers to discriminate naturalistic textures. The spike rate of each Izhikevich neuron was used as the input feature of the classifiers. Two classifiers (Random Forests and KNN) were used. For classification, fivefold cross-validation was utilized. The data samples were divided into 5 subgroups. Each time, one of these 5 subgroups was used as the test set and the remaining 4 subgroups formed the training set. Finally, the average performance across all 5 trials for each subset was computed and reported in Fig. 5. For the Random Forests classifier, the number of estimators was set to 1000. The results are illustrated in Fig. 5a for the spike rate algorithm^10,36^. The synergy between muscle spindle and skin mechanoreceptors shows better texture discrimination than the collaboration between SA-I and RA-I afferents across scanning speeds (Fig. 5a). It is worth noting that the MS-Prim has a significant contribution compared to the MS-Sec in encoding fascicle speeds in agreement with the biological evidence^9^ (Fig. 5a**)**. Moreover, the synergy between PC fibers and SA-I/RA-I leads to the higher accuracy (82%) by encoding more information about texture characteristics. Acceptable classification accuracy was obtained with responses of SA-I/RA-I (72% accuracy), in Fig. 5b. However, the synergy between all receptors resulted in 92% accuracy (Fig. 5c) in recognizing 10 naturalist textures across 6 different scanning speeds.

Next, we evaluate the proposed multi-channel neuromorphic system to determine how much the robotic finger should explore the surfaces to distinguish the textures. Figure 5d shows that the biomimetic fingertip in conjunction with the proprioception sensor encodes enough tactile and motion information only with 10 mm of surface exploration (displacement), and afterward, the results almost remain constant. This ability enables robots to recognize textures quickly and touches only a small part of the surface. The spike rate feature was again used as the input of the Random Forests and KNN classifiers. Figure 5d shows that the Random Forests has better performance again. Similar to the biological reports^41^, in Fig. 5e, the response of the Pacinian fiber (RA-II) indicates speed sensitivity during exploring three fine textures across the highest (90 mm/s) and the lowest (40 mm/s) scanning speeds. In the other words, increasing scanning speed leads to an increase in the firing rate of MS-Prim and rapidly adapting tactile afferents (RA-I/RA-II). However, MS-Sec and SA-I responses remain almost constant due to being low sensitive to the velocity and also manipulating fine textures.

## Conclusion

Multi-finger robotic hands serving as prosthetic hands equipped with tactile sensory feedback can increase the safety of object manipulation and the accuracy of texture recognition in amputees. Indeed, integration of multiple sensory feedback incorporated into prosthetic hands should be helpful in dealing with the external environment, slip detection, and fine motor activities. The inspiration from human and animal sensory systems facilitates the proposing of efficient methods for dealing with sophisticated problems such as robotics/prosthetics applications. In such cases, our proposed hydro-elastomer sensor can be easily placed on the robot to provide proprioceptive feedback as replicating muscle spindles. Moreover, this biomimetic fingertip comprises two high-performance tactile sensors to mimic the three types of human skin mechanoreceptors. This sensor integration helps to have accurate tactile and motion perception in daily tasks and real-time object manipulation. Our results show that the integration of two types of tactile sensors with proprioception sensors can recognize different textures under variable scanning speeds without any high computational cost. A robotic/prosthetic hand with this kind of multiple sensory capabilities to encode different textures can operate under different scanning speeds. The proposed bio-inspired system discriminates all 10 fine naturalistic textures (S1-S6 in Materials and Methods section) with 6 different scanning speeds. In this research, the spike rate algorithm was used which simply considers the number of produced spikes normalized to the time. Although other features such as Victor–Purpura distance and van Rossum distance can also be used which may improve the classification performance due to providing more information based on the time of spikes. Note that the contact force can be extracted from the 8 × 8 pressure sensor data (the DC level), and the previous study showed that the SA-I afferents respond to the applied contact force for sharpness recognition^36^. This synergy in sensory feedback showed better performance and classification accuracy under different scanning speeds compared to the previous studies as illustrated in Table 1. This table demonstrates the sensors and methods utilized in related research, including this research. Our system proved better results in spite of being tested in more variable palpation speeds due to the integration of several sensory organs rather than commonly-used skin mechanoreceptors alone.

**Table 1:** Tactile sensing using neuromorphic methods. (MR: Mechanoreceptor).

Author	Yi, et al.^10^	Gupta, et al.^43^	Oddo. et al.^16^	Sankar, et al.^19^	Rongala, et al.^14^	Friedl, et al.^44^	Liu, et al.^15^	This work
Tactile sensor type	2 Piezoelectric	4 × 4 piezoresistive	A 2 × 2 array of four MEMS micro sensors	3 × 3 Piezoresistive	4 × 4 Piezoresistive	2 Piezoelectric, 1 Piezoceramic	4 × 4 Piezoelectric	8 × 8 Piezoresistive and Piezoelectric
MRs’ density (unit/cm^2^)	–	10	72	8	72	-	25	25
Applications	Roughness discrimination	Texture discrimination	Roughness discrimination	Texture discrimination	Texture discrimination	Texture discrimination	Roughness discrimination	Texture discrimination
Mechanoreceptor	Meissner	Merkel	Merkel	Merkel	Merkel	Meissner, Merkel, Pacinian,	Pacinian	Meissner, Merkel, Pacinian, Muscle spindles
Spike train classification	KNN	KNN	KNN	SVM	KNN	SVM	-	KNN, Random forests
Innervation technique	–	–	–	–	–	–	–	** + **
Scanning speed independence Method	–	–	The phase difference between two adjacent taxels	–	Neural network	–	Principal frequency of vibration, The response time interval between taxels	Data fusion of tactile mechanoreceptor and muscle spindles

In Table.1, four methods are presented which are independent of scanning speed during roughness and texture discrimination. Oddo and his colleagues^16^ proposed a method based on the phase difference between two adjacent taxels. Liu, et al.^15^ presented a speed-independent roughness discrimination method by analyzing the principal frequency of vibration and response time interval between two adjacent taxels. These two studies have presented valuable methods for discriminating stereotyped textures with known and predetermined dimensions. Rongala and his collaborators^14^ have employed a two-layer spiking neural network for discriminating textures under different scanning speeds. However, their systems did not provide any velocity feedback and sensor integration. Nevertheless, our work presents a method that leads to stable texture discrimination under varying scanning speeds for naturalistic textures with unknown dimensions and spatial details. Indeed, tactile sensors in conjunction with proprioception sensors can increase the performance of texture recognition by providing information about the speed and position of the joints. In spite of having less density of sensing elements compared to the reports of Rongala et al.^14^, our work included multisensory integration, complex mechanoreceptors’ receptive fields, and classification methods that resulted in accurate texture discrimination for variable palpation speeds. It should be pointed out that similar to previous studies^19,36,42^, cross-talk occurs because of reading data in row and column configuration which brings some undesired resistive paths during manipulations. Indeed, some reading can be registered in a corner of a square sensor when the force is applied in the opposite corner, and thus the contact is not local which may affect the receptive fields. Nevertheless, this effect can be compensated to some extent due to presence of learning. Finally, this sensory system can be implemented on the robotic and prosthetic hands with deep learning methods to have accurate texture recognition in real applications. These findings motivate employing the proposed architecture for sensor integration in neuromorphic tactile devices.

## Material and methods

The objectives were to show that (i) innervating an 8 × 8 tactile sensor with artificial afferents, (ii) proposing a biomimetic fingertip that is capable of identifying textures and contact forces simultaneously by the implementation of the skin’s mechanoreceptors, and (iii) recognition of the scanned textures with variable speeds through the integration of proprioception sensor and biomimetic fingertip.

### Experimental setup

**Figure 6:**
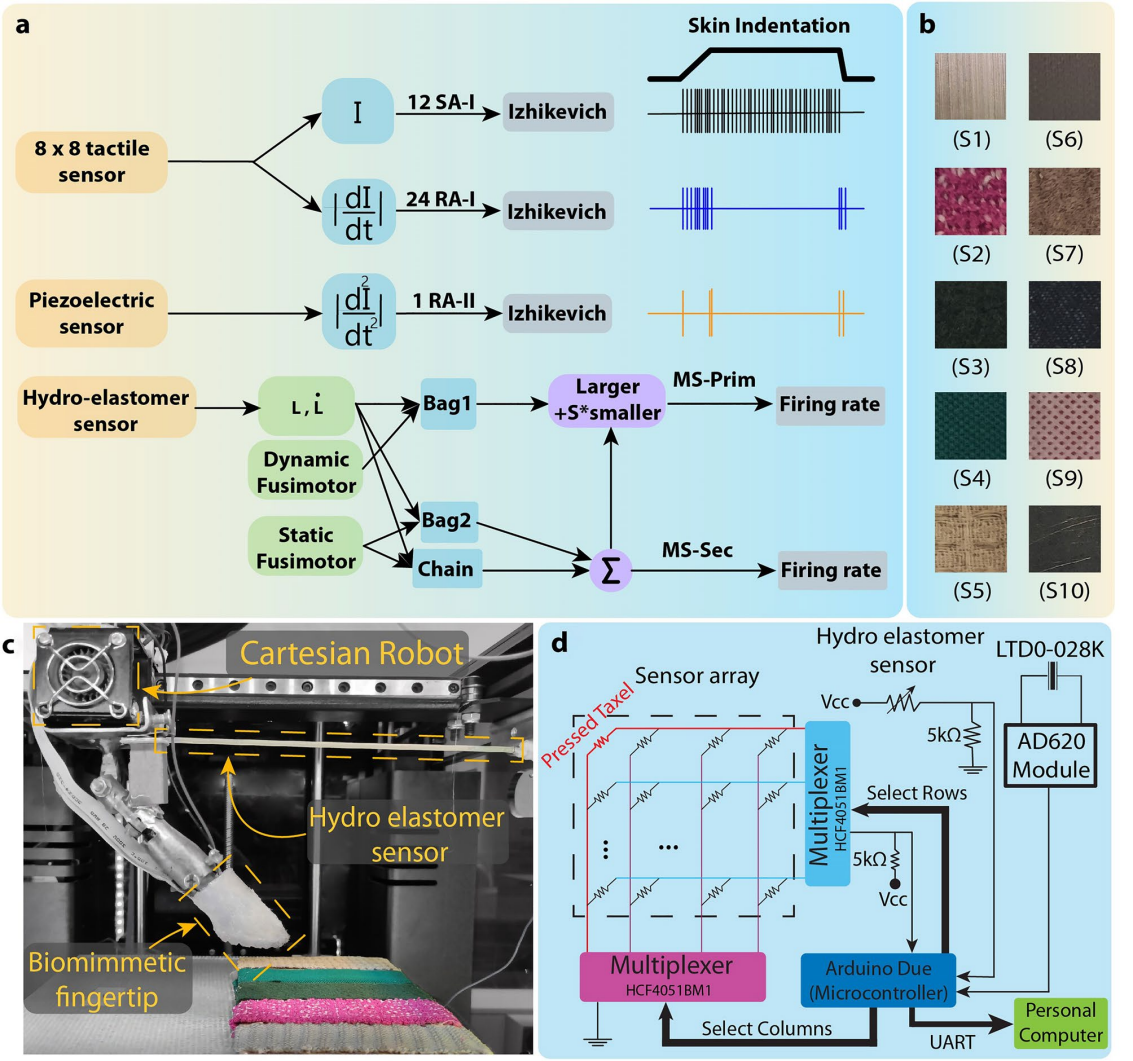
The illustration of the experimental setup comprises a robotic system with three degrees of freedom, three tactile sensors, and naturalistic textures. (**a**) Schematic of all preprocessing stages and converting recorded data to the appropriate input current to be applied to the Izhikevich neural model to produce spike trains. (**b**) Ten different naturalistic fine textures. (**c**) The experimental setup with the custom-built robot and sensors. A hydro-elastomer sensor to mimic muscle spindle (a proprioception sensor) for encoding the speed and position of the biomimetic finger. An 8 × 8 pressure tactile and piezoelectric sensors were embedded within a soft biomimetic fingertip to encode texture details during manipulation. (**d**) Scheme of the interface circuit for data collection.

A multi-channel neuromorphic tactile system (Fig. 6a) was implemented that includes a 3 DOF cartesian robot and 3 different sensors, namely, an 8 × 8 piezoresistive tactile, a piezoelectric, and a hydro-elastomer sensor (Fig. 6c). The proposed biomimetic fingertip is mounted on a 3 DOF Cartesian robot with position control. Ten fine naturalistic textures (S1-S10) were prepared with everyday materials (not regular textures with gratings and printed dot patterns) to investigate the effect of scanning speed on the recognized textures (Fig. 6b). The biomimetic finger scans 10 naturalistic textures with 6 different speeds ranging from 40 to 90 mm/s. Every single object was scanned ten times. The hydro-elastomer sensor is fixed to one side of the static wall, and the other side is connected to the cartesian robot. The robot was controlled by a Python code (Python 3.9.0, Data availability). The supplementary video shows the experimental setup and process of the data collection (Additional Information).

### Hydro-elastomer sensor fabrication

The hydro-elastomer sensor was made by the following steps: (i) We cut a stretchable elastomer silicone rubber tube with good elasticity, transparency, and high durability to the desired dimensions. (ii) Then, the silicone tube was filled with a commercial conductive hydrogel. Hydrogel is a commercial electrode gel that is used for recoding of ECG (electrocardiogram) signals, which has been chosen because of being accessible and inexpensive. (iii) After the hydrogel injection, each side of the tube was fastened by copper electrodes. The manufactured proprioception sensor was made with available, off-the-shelf materials and was highly stretchable, lightweight, and inexpensive.

### Fabrication of biomimetic fingertip

The 8 × 8 tactile sensor array has 64 taxels (sensing elements) to convey contact force and spatial information about the textures. The sensor’s taxels can be easily scaled up based on the desired application. The tactile sensor was fabricated using a commercial pressure-sensitive sheet (Velostat/Linqstat), whose resistance is reduced as a result of applying force to the material. A piezoresistive sheet was sandwiched between two layers of flexible printed circuit boards (PCB) conductive traces as rows and columns layers. The tactile sensor has 8 rows and 8 columns arranged with a 1.5 mm pitch (16 mm × 16 mm active area). The sensor was embedded within a silicone layer of the fingertip to disperse the indentation force and mimic Meissner and Merkel mechanoreceptors. The sensor array had 64 taxels with a total active sensing rectangular area of 256 mm^2^. It should be pointed out that this configuration in rows and columns provides undesired resistive paths, which introduces crosstalk, and thus choosing suitable interface circuit is necessary to reduce this effect. The biomimetic fingertip was constructed as follows: (i) A cuboid hard bar with 8 mm × 8 mm × 50 mm dimensions is obtained by cutting a PVC sheet to mimic human finger bone. According to the higher hardness of the epidermis than the dermis^10^, a commercial sensor LTD0-028 K was used and arranged on the top of the bar to mimic the Pacinian corpuscle, which is close to the finger bone in the deeper layer of skin (dermis) and is sensitive to high-frequency vibrations. (ii) By altering the mixing ratio of base to agent PDMS, two PDMS layers with different hardness were obtained to emulate the epidermis and dermis layers of human skin (at 85 °C for 30 min of curing). (iii) The 8 × 8 tactile sensor was inserted into a 3D printed mold with a size comparable to the human fingertip, to be placed in the outer layer of the biomimetic fingertip (closer to the epidermis) to mimic the Meissner and Merkel mechanoreceptors. Then the mold was filled with the harder PDMS. Next, the bar with a PVDF film sensor was inserted into the mold and filled with the softer PDMS. When the piezo is displaced from the mechanical neutral axis, it generates high voltages as a response to the vibrations.

### Interface circuit

Two analog multiplexers and an Arduino Due microcontroller with a 12-bit ADC were used as the readout circuits with 12 analog inputs. To reduce the cost of the interface circuit, a bidirectional analog multiplexer with 8 independent inputs and 1 output (8:1) was used to read each common line of the 8 × 8 sensor. Each taxel was at the intersection of the rows and columns of PCBs’ electrodes, and multiplexers were connected to each row and column. The output of one of the multiplexers was connected to the ground and the other was connected to 5 V with a variable resistor (for changing the sensor's sensitivity). One specific taxel was selected by selecting each column and row with a microcontroller and a voltage-divider circuit. The piezoelectric and hydro-elastomer sensors were connected to the microcontroller through an AD620 amplifier module and voltage divider circuit (Fig. 6d). Then the voltage response of each taxel, piezoelectric and hydro-elastomer sensors were sampled by the ARM microcontroller at 1 kHz and processed in Python code. The innervation technique was implemented in the ARM microcontroller to create the complex receptive fields^36^. The interface circuit for data collection was shown in Fig. 6d.

### Data preprocessing

The interface circuit converts the output of the sensors to digital signals and transmits them through serial communication with a baud rate of 115,200 bps to a PC for further processing in Python. The piezoelectric data were normalized and band-pass-filtered between 5 and 250 Hz using an eighth-order Butterworth filter to remove unwanted robot motor noise and the DC voltage before injecting it as the input of the Izhikevich neuron model (described below). Also, the recorded data of the hydro-elastomer sensor was filtered through a high-pass filter with a 1 Hz cutoff frequency and first order Butterworth filter to remove the noise. The hydro-elastomer data was mapped between 0.95 and 1.08 to be suitable as the input of the muscle spindle model.

### Neuromorphic encoding

Encoding spatial and temporal information is essential when processing dynamic stimuli such as textures. The Izhikevich neuron model was used to mimic the SA-I, RA-I, and RA-II tactile afferents' activities. According to the previous research, the SA-I and RA-I afferents ratio is 1:2^36,45,46^ and can be scaled up for the desired purpose. The Izhikevich model is described as follows;

1 $$v\prime = 0.04v^{2} + 5v + 140 - u + G\frac{I}{{C_{m} }}$$

2 $$u\prime = a\left( {bv - u} \right)$$

3 $$if\;v \ge 30\;mV \to then\left\{ {\begin{array}{*{20}l} {v \leftarrow c} \hfill \\ {u \leftarrow u + d} \hfill \\ \end{array} } \right.$$

*v* and *u* are the membrane potential of the neuron and the membrane recovery variable, respectively. *I* is the input current. *a, b, c*, and *d*, are the constant neuron parameters. *G* is the gain factor that has a specific value for each type of mechanoreceptor, as listed in Table 2. *C*_*m*_ is the capacitance value for dimensionality consistency and is equal to 1 F. In Table 2, the Izhikevich model parameters are shown which have been taken from^40^.

**Table 2:** Parameter values of the Izhikevich neuron model in regular spiking mode.

Parameter	Values
a	0.02 s^−1^
b	0.2 s^−1^
c	−65 mV
d	8 mV
G = G_SA-I_	1.3 kΩ
G = G_RA-I_	17 kΩ
G = G_RA-II_	2 kΩ

### Muscle spindle model

Different mathematical models of muscle spindles have been developed previously^9,32^. The muscle spindle model proposed by Mileusnic et al.^32^, includes fusimotor activation, primary and secondary afferent activities, and is suitable for implementation in closed-loop control systems. This model consists of three intrafusal fibers (Bag1, Bag2, Chain) with the same function and different parameters. The function has two types of inputs: the fascicle length L (and its derivatives $\dot{L}$) and the relevant fusimotor activation level (f_dynamic_, f_static_). Vannuci et al.^9,47^ modified this model to reduce the unnecessary complexities and adapted it for real-time neuromorphic applications. In this paper, we used Vannuci’s model. The firing rate of MS-Prim afferent is a combination of all intrafusal fibers’ activities which consists of Bag1 fiber and the sum of Bag2 and Chain responses (Fig. 6a). Moreover, the smaller response between Bag1 and the sum of Bag2 and Chain, is multiplied by S = 0.156. Next, the sum of all fibers is used as the response of MS-Prim afferent as illustrated in Fig. 6a. MS-Prim carries information to the central nervous system that depends on both the length and stretch speed of the muscle, while MS-Sec consists of only the sum of Bag2 and Chain fibers’ responses which provide information mostly about the position (more details in^9,32,47^). The fascicle length and the rate of its changes (L and $\dot{L}$) are given by the hydro-elastomer sensor during every surface exploration with mapping the maximum and minimum sensor values to 0.95 and 1.08, respectively. In addition, the fusimotor activation levels of efferent are given to the model as the constant inputs during each experiment. Every stretch was repeated under specific fusimotor drives ($f$_dynamic_ = 70 spikes/s and $f$_static_ = 70 spikes/s)^9^.

## Supplementary Information

Supplementary Information 1.

Supplementary Video 1.

## Data Availability

All data are available from the corresponding author upon reasonable request.

## Code availability

All analyses reported in this work were made with custom code written in Python 3.9, and will be available from the corresponding author upon reasonable request. Moreover, the last version will be uploaded at: https://github.com/Research-lab-KUMS/Texture-recognition.
