# Supplementary material for: Texture recognition based on multi-sensory integration of proprioceptive and tactile signals
Source: Sci Rep. 2022 Dec 15;12:21690. doi: 10.1038/s41598-022-24640-5 (PMC9755227; doi:10.1038/s41598-022-24640-5)
Supplement: Supplementary file 1 — Supplementary Information 1. [file 41598_2022_24640_MOESM1_ESM.docx]

**Texture recognition based on multi-sensory integration of proprioceptive and tactile signals**

**Behnam Rostamian ^1^, MohammadReza Koolani ^1^, Pouya Abdollahzade ^1^, Milad Lankarany ^2,3^, Egidio Falotico ^4^, Mahmood Amiri ^5^*, Nitish V.Thakor ^6,7^**

**1** Medical Biology Research Center, Institute of Health Technology, Kermanshah University of Medical Sciences, Kermanshah, Iran

**2** Krembil Research Institute – University Health Network (UHN), Toronto, Ontario, Canada

**3** Institute of Biomedical Engineering & Department of Physiology, University of Toronto, Toronto,

**4** The BioRobotics Institute, Scuola Superiore Sant’Anna, Pontedera, Italy

**5** Medical Technology Research Center, Institute of Health Technology, Kermanshah University of Medical Sciences, Kermanshah, Iran

**6** Department of Biomedical Engineering, Johns Hopkins University, Baltimore, MD, USA

**7** Department of Biomedical Engineering, National University of Singapore, Singapore.

***** corresponding author,

Address: Medical Technology Research Center, Parastar Ave., Kermanshah, Iran

Email: [ma_amiri_bme@yahoo.com](mailto:ma_amiri_bme@yahoo.com)

Tel: +989183567802

**Additional information**


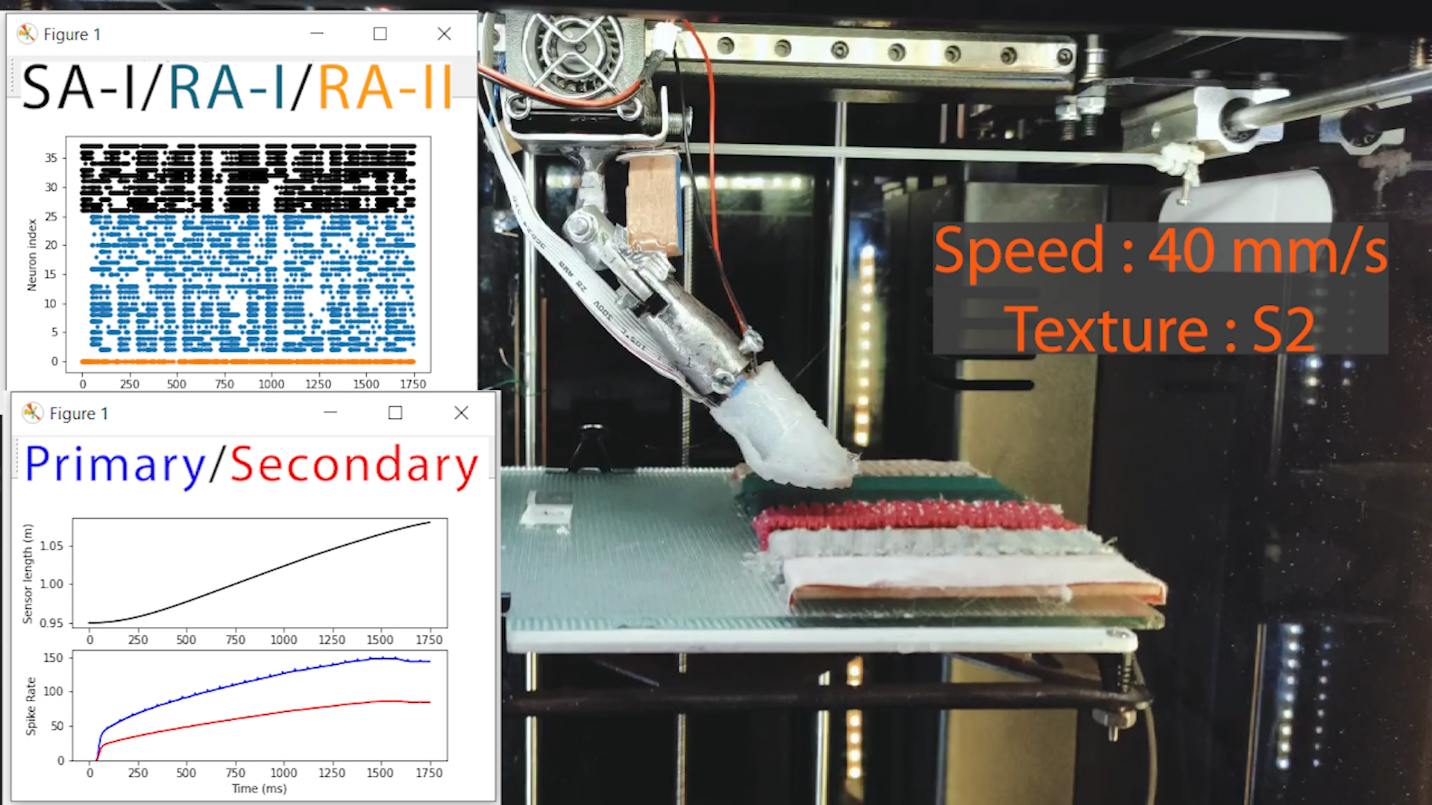


**Movie S1** –The experimental setup and process of the data collection. In this video, the firing patterns of SA-I, RA-I, RA-II, primary and secondary afferents of the muscle spindle are shown. The biomimetic finger equipped with a tactile sensor (8*8 taxel array), a piezoelectric sensor, and a hydro-elastomer sensor, was installed on a 3 DOF Cartesian robot with position control. The biomimetic finger scans 10 different naturalistic textures with six different scanning speeds.
